# “Good Epidemiology Practice” Guidelines for Pesticide Exposure Assessment

**DOI:** 10.3390/ijerph17145114

**Published:** 2020-07-15

**Authors:** Julie E. Goodman, Robyn L. Prueitt, Paolo Boffetta, Crispin Halsall, Andrew Sweetman

**Affiliations:** 1 Gradient, One Beacon Street, 17th Floor, Boston, MA 02108, USA; 2 Gradient, 600 Stewart Street, Suite 1900, Seattle, WA 98101, USA; rprueitt@gradientcorp.com; 3 Stony Brook Cancer Center, Department of Family, Population and Preventive Medicine, Stony Brook University, Stony Brook, NY 11794, USA; paolo.boffetta@gmail.com; 4 Department of Medical and Surgical Sciences, University of Bologna, 40126 Bologna, Italy; 5 Lancaster Environment Center, Lancaster University, Lancaster LA1 4YQ, UK; c.halsall@lancaster.ac.uk (C.H.); a.sweetman@lancaster.ac.uk (A.S.)

**Keywords:** epidemiology, methodology, exposure assessment, pesticides

## Abstract

Both toxicology and epidemiology are used to inform hazard and risk assessment in regulatory settings, particularly for pesticides. While toxicology studies involve controlled, quantifiable exposures that are often administered according to standardized protocols, estimating exposure in observational epidemiology studies is challenging, and there is no established guidance for doing so. However, there are several frameworks for evaluating the quality of published epidemiology studies. We previously developed a preliminary list of methodology and reporting standards for epidemiology studies, called Good Epidemiology Practice (GEP) guidelines, based on a critical review of standardized toxicology protocols and available frameworks for evaluating epidemiology study quality. We determined that exposure characterization is one of the most critical areas for which standards are needed. Here, we propose GEP guidelines for pesticide exposure assessment based on the source of exposure data (i.e., biomonitoring and environmental samples, questionnaire/interview/expert record review, and dietary exposures based on measurements of residues in food and food consumption). It is expected that these GEP guidelines will facilitate the conduct of higher-quality epidemiology studies that can be used as a basis for more scientifically sound regulatory risk assessment and policy making.

## 1. Introduction

Good Laboratory Practice (GLP) guidelines are a set of principles for the planning, performance, monitoring, recording, reporting, and archiving of non-clinical laboratory studies. The intention of GLP guidelines is to ensure the quality, reliability, and integrity of scientific research. The United States Environmental Protection Agency (US EPA) monitors compliance with GLP guidelines for all test data submitted to the Agency in support of pesticide product registration, as required by the Federal Insecticide, Fungicide, and Rodenticide Act (FIFRA) [1].

While there are several frameworks for assessing epidemiology studies (e.g., LaKind et al. [2], NTP [3], von Elm et al. [4]), unlike toxicology studies, there are no universal standards like GLP for developing epidemiology study protocols. This is disconcerting because epidemiology evidence has recently factored more prominently in regulatory hazard and risk assessments of pesticides and other chemicals [5,6].

Epidemiology studies are generally observational in nature, and there are several epidemiology study designs. For example, in case-control studies, individuals with (cases) and without (controls), a specific disease are identified, and then exposure is measured or estimated in each person [7]. In cohort studies, a cohort, or group of individuals who share a common characteristic (e.g., place of residence or occupation), is followed over time, and individual exposures and diseases are documented [8,9]. In cross-sectional studies, individual exposure and disease status are ascertained together at one point in time or over a short, defined period [8,9].

Observational epidemiology studies of pesticide exposures can be useful for generating new hypotheses, but many studies are not informative for inferring causation between pesticide exposures and potential diseases or other health effects due to various critical methodological limitations. For example, the lack of temporality (i.e., exposures are not measured or estimated prior to the outcome) in cross-sectional studies means that these studies cannot inform causation. Another example is that in case-control studies, recall bias can have a substantial impact on exposure assessment and can sometimes lead to spurious associations. To assess causation, any epidemiology study investigating health effects or diseases associated with a particular exposure should test one or more biologically plausible hypothesis and should define a priori outcomes that are consistent with the evaluated hypothesis. In addition, epidemiology studies must be of sufficient quality to inform causal determination and quantitative risk assessment, two key elements in pesticide regulations [10]. For causal determinations, studies need to establish the presence of an effect following an exposure. For quantitative risk assessment (dose-response analysis), studies need to not only establish the presence of an effect, but also the magnitude of an effect in relation to the level of the exposure.

There have been various efforts in recent years to develop “Good Epidemiology Practice” (GEP) guidelines for improving the quality of epidemiology research [11,12,13] and protocols to aid in specific aspects of pesticide exposure and risk assessment [14,15,16], but GEP guidance for pesticide research is not available. We developed a preliminary list of considerations for GEP for environmental epidemiology studies; these are based on various study quality guidelines and frameworks (Table 1). Some of these considerations are applicable to all study designs, while others are tailored to certain designs. Of the considerations in this list, we determined that exposure characterization is one of the most critical areas for which standards are needed in epidemiology studies of pesticide exposure. As such, while the goal is to eventually develop a larger, complete set of GEP guidelines for all phases of the design, conduct, and reporting of epidemiology studies of pesticides, this paper is specific to detailed guidance for exposure characterization.

## 2. Materials and Methods

We developed this guidance based on a review of numerous existing guidance and regulatory documents that aim to improve the quality and reporting of a variety of different types of evidence, including from human, animal, and in vitro studies. This includes the exposure characterization the US EPA Office of Pesticide Programs (OPP) discusses in “Framework for Incorporating Human Epidemiologic and Incident Data in Risk Assessments for Pesticides” [10], and GLP documents for toxicity studies (primarily WHO [17]) and pesticide residues [18]. We also considered existing post hoc study quality assessment systems—specifically, the US EPA Integrated Risk Information System (IRIS) Risk of Bias (RoB) framework and the National Toxicology Program (NTP) Office of Health Assessment and Translation RoB tool, as well as the LaKind et al. [2] BEES-C tool, peer-reviewed articles on exposure assessment methods for epidemiology, and our own research on exposure characterization and study-quality evaluation methods (see for example, Goodman et al. [19] and Lynch et al. [20,21]). Combining overarching principles and specific guidance from each of these documents, tailored where possible specifically to pesticides, we developed the GEP criteria for exposure assessment with requirements for the design, conduct, and reporting of studies that can inform causation and quantitative risk assessment.

## 3. Proposed GEP Guidelines for Pesticide Exposure Assessment

We organized guidelines for pesticide exposure assessment based on the type of data available. Biomonitoring and environmental sampling involve direct measurements of pesticides, their metabolites, or other biomarkers. In contrast, questionnaires and interviews provide different types of exposure data, such as information on the presence, duration, or frequency of exposure. Sampling and interview data may be used for all exposure routes (i.e., dermal, ingestion, and inhalation) and most types of exposures, including those in occupational (e.g., farmhands, sprayers, pest control professionals, greenhouse workers, and chemical manufacturers) or non-occupational (e.g., residential pesticide users and individuals who live or work near sprayers) settings. However, they are not sufficient for addressing dietary exposures to pesticides, as this involves measuring the concentrations of pesticide residues in foods and assessing the consumption of these foods.

Thus, the proposed GEP guidelines for pesticide exposure assessment, as presented in Table 2, Table 3 and Table 4, are based on the sources of exposure data that share common requirements (i.e., biomonitoring and environmental samples, questionnaire/interview/expert record review, and dietary exposures based on measurements of residues in food and food consumption). These guidelines primarily apply to exposure assessment in both cohort and case-control studies, for which, unlike cross-sectional studies, temporality between the exposure and the outcome can be established. Some specific criteria are noted to differ by study design, however. The criteria also apply to exposures to both single substances and complex mixtures. Most criteria apply to both causal inference and quantitative risk assessment, and we specify with underlined text in the tables where additional requirements are needed for quantitative risk assessment.

### 3.1. Biomonitoring and Environmental Sampling

Table 2 presents the criteria for estimating pesticide exposures with biomonitoring (e.g., urine, blood), personal exposure monitoring (e.g., dermal wipes or washes, breathing zone air sampling) or environmental sampling data (e.g., surface wipes, ambient air monitoring). Biomonitoring integrates exposures from different routes to quantify the amount of a substance absorbed by the body, whereas personal exposure monitoring characterizes exposure at the point of entry into the body [10]. Biomonitoring and personal exposure monitoring are generally considered the best sources of data for estimating actual exposure concentrations, though they are often conducted over a limited time period that may not be sufficient to accurately reflect longitudinal patterns of exposure [10]. Environmental sampling characterizes substance concentrations in environmental media and is useful for estimating exposures defined by geographical boundaries (such as in ambient air and drinking water), but can be less reliable for assigning individual-level exposures [10].

Some criteria in Table 2 are general, while others are specific to biomonitoring, personal monitoring, or environmental sampling data. The categories of criteria include study protocol; the validity and reliability of sampling; exposure window; time integration, specificity, and sensitivity of the exposure metric; the validity and reliability of the analytical methods; sample replicates; consideration and adjustment of matrix effects; sample storage and stability; and reporting requirements.

### 3.2. Participant Questionnaires and Interviews

Table 3 presents the criteria for estimating pesticide exposures with questionnaires, interviews, and/or expert record review. Such methods are typically used to assign categorical levels of exposure that are surrogates for actual exposure levels [10]. The categories of criteria for these methods include study protocol, exposure window, study population, the design of self-administered and interview questionnaires, the blinding of study participants and investigators, validation of exposure assessment methods, and reporting requirements.

### 3.3. Dietary Exposures

Table 4 presents the GEP criteria for assessing dietary exposures to pesticide contamination or residues in food. The assessment of dietary pesticide exposures requires two components: measuring the concentrations of pesticide residues in foods and assessing the consumption of these foods. Separate criteria are presented for these two components. The former component warrants similar criteria to those for environmental sampling, with a few specific categories modified to be applicable to foods. The latter component generally requires the use of questionnaires, interviews, or food diaries. On the basis of the criteria presented in Table 3, the criteria for this component are tailored towards assessing food consumption.

## 4. Discussion

The goals of epidemiology studies vary, and while many are useful for hypothesis generation, they are not all informative for inferring causation. Unlike toxicity studies in laboratory animals, for which the exposures are well characterized and a set of GLP guidelines are available, there are no such guidelines (to the authors’ knowledge) available to ensure the quality and reliability of exposure assessment in epidemiology studies. Regardless, epidemiology studies are being used more often in risk assessment, and while many organizations have proposed criteria on which to judge the quality and reliability of epidemiology studies (e.g., see Lynch et al. [21]), there are no standard guidelines, such as GLP, that can be used to ensure the reliability and appropriateness of epidemiology study results for risk assessment.

We proposed GEP criteria for pesticide exposure assessment, for each of the three general types of data available (biomonitoring and environmental sampling; questionnaires and interviews; and dietary exposures). Although the proposed criteria could be subdivided further by data type or study design, they are intended to represent a balance between widely-applicable guidelines that are flexible enough to be applied to many different chemicals and research questions and more specific guidance that details expectations for different study designs and exposure data. Our criteria have a greater level of detail than existing guidance (e.g., the OPP epidemiology framework; [10]) and existing study quality evaluation systems (e.g., see Lynch et al. [21]), but are intended to be concise enough to maintain clarity and ease of compliance.

We note that, similar to GLP guidelines, we did not include scores that align with the importance of each category of GEP criteria. While all categories are important, study results may still be reliable if all categories are not met. For example, even if sample storage and stability are not verified, it is possible that both are sufficient. However, we do believe several categories are critical and must be met for a study to be used in risk assessments. In studies where exposures are measured, the validity and reliability of sampling and analytical methods must be confirmed, and sensitivity and specificity must be considered. When exposure information is ascertained by records or interviews, these methods must also be validated. If this is not carried out, then it is difficult to determine the reliability of exposure estimates. If exposure is not reliable, then the results are uncertain and open to question. Unreliable exposure estimates can lead to exposure misclassification, which generally refers to the incorrect assignment of participants to categories of exposure (e.g., low and high), or exposure measurement error, which generally refers to errors with measures of exposure on a continuous scale.

Exposure misclassification can be either differential (i.e., misclassification differs between exposed and unexposed groups) or non-differential (i.e., all groups have an equal likelihood of being misclassified) [36]. Although it is often stated that non-differential misclassification always biases effect estimates toward the null, both types of misclassification can bias effect estimates in either direction [37].

In its recent draft position paper on the use of epidemiology studies in pesticide risk assessment and management, the European Food Safety Authority (EFSA) Panel on Plant Protection Products and their Residues (PPR) noted:

While it is commonly assumed by some that non-differential misclassification bias produces predictable biases toward the null (and thus systematically under-predicts the effect size), this is not necessarily the case. Also, the sometimes-common assumption in epidemiology studies that misclassification is non-differential (which is sometimes also paired with the assumption that non-differential misclassification bias is always toward the null) is not always justified (e.g., see Jurek et al. 2005. [38]).

In fact, several quantitative analyses have demonstrated realistic scenarios under which approximately non-differential exposure measurement errors can bias results away from the null [36,39,40]. For example, Jurek et al. [36] showed that associations measured in datasets with low exposure prevalence are especially vulnerable to exposure misclassification that is nearly, but not completely, non-differential. For quantitative risk assessment, the direction and magnitude of exposure measurement error/misclassification and the impact on results should be evaluated quantitatively through simulations and sensitivity analyses. While exposure measurement error/misclassification may not ever be entirely eliminated, conducting exposure assessments using GEP will help minimize the impact of this bias.

As the proposed guidelines in this paper are specific for exposure assessment, we plan to develop similar detailed guidelines for other aspects of epidemiology studies. However, the aspect of confounding bears mentioning here, as many potential confounders will be measured in the same way as the exposures of interest and should be subject to the same criteria for assessment. For example, certain potential confounders (e.g., body mass index [BMI], smoking, and alcohol consumption) can be measured by questionnaire and/or interviews, whereas others (e.g., co-exposures to other pesticides or chemicals) can be measured through biomonitoring or environmental sampling. Thus, some GEP guidelines specific to the aspect of confounding would also be relevant to those for exposure assessment.

These guidelines should be used to design epidemiology studies moving forward and also to evaluate studies conducted in the past so that it can be determined how the results of these studies should be considered in a regulatory setting (e.g., how they contribute to the weight of evidence regarding causation, and whether and how they should be used in quantitative risk assessment or for comparing human exposures to doses in animal toxicity studies). The more criteria a study satisfies, the more robust the study quality and results will be. Although studies that do not fulfill these criteria should not be used for causal inference or quantitative risk assessment, they may still be important for generating new hypotheses and can contribute considerably to advancing the science.

## 5. Conclusions

It is expected that the GEP guidelines proposed in this paper will facilitate the conduct of higher-quality epidemiology studies that can be used as a basis for more scientifically sound regulatory risk assessment and policy making.

## Figures and Tables

**Table 1:** Requirements for Conducting High-Quality Epidemiology Studies.

Category	#	Requirement
Objectives and Study Plans	1	Clearly state study objectives
	2	Review ethical guidelines for human research
	3	Create and follow an a priori study protocol
	4	Write and follow a Standard Operating Procedure (SOP)
	5	Write and follow a quality assurance plan
	6	Describe all deviations from study protocol
	7	Provide dates of study initiation and completion
	8	Document roles and responsibilities of all staff
Study Design	1	Report study design and setting (e.g., date, location)
	2a	Document participant selection process and participant inclusion/exclusion criteria
	2b	Use consistent participant recruitment methods to ensure that baseline differences between groups are minimized
	3	Report participant characteristics (e.g., age, sex)
	4a	Report study size
	4b	Use a sufficient study size so that estimates are not subject to a large degree of imprecision
	4c	Conduct and report study power analysis
	5	Blind outcome assessors to exposure status
	6a	Report participation rate/attrition
	6b	Maintain similar attrition rate across groups
	6c	Minimize loss to follow-up/ensure a high response rate
	6d	Discuss the potential for selection bias
	7a	Provide detailed discussion of comparison groups
	7b	Ensure that comparison groups are similar to cases/exposed subjects
Exposure Characterization	1	Choose exposure metric in outcome-relevant time window
	2	Report exposure levels and units of measurement
	3	Use a measurement that is sensitive, valid, and applied consistently ^a^
	4	Assess exposure independent of outcome
	5	Assess and report the potential for exposure measurement error/misclassification
	6	Report the stability and storage of biological samples, as applicable
	7	Describe QA/QC procedures, limits of detection or quantification, standards recovery, measures of repeatability, and investigation and prevention of contamination through appropriate use of blanks
	8	Ensure that there are a sufficient number of samples (to be statistically meaningful for the investigation) or, in the instance of a single sample, evidence that errors are negligible
Outcome Assessment Methods	1a	Provide detailed statistical methods
	1b	Use appropriate statistical techniques
	1c	Document all calculations and analyses
	2	Report all data sources (including raw data upon request of regulator)
	3	Report data measurement methods
	4	Perform and report QA/QC methods for outcome measurement
	5	Provide collection date and signature of person entering data
	6a	Use validated outcome assessment methods
	6b	Discuss potential for outcome misclassification
	7a	Identify sources of bias and confounding a priori based on hypothesis being tested
	7b	Report how confounding and bias are addressed
	7c	Control for co-exposures
	7d	Reduce possibility for bias through design ^b^
Study Results	1a	Report the results of all measured outcomes and adjusted and unadjusted analyses (i.e., complete outcome reporting)
	1b	Report the results of sensitivity, subgroup, or other analyses
Discussion	1	Report study limitations
	2	Provide an interpretation of results
	3	Discuss issues of generalizability
	4a	Report the funding source/provide a Conflict of Interest statement
	4b	Discuss whether the funding source affected design or interpretation
	5	Discuss other sources of bias

# = Numbering for the list of requirements in each category; QA/QC = Quality Assurance/Quality Control; ^a^ That is, use well-established, validated, quantitative exposure assessment methods at the individual level, with as little measurement error as possible; ^b^ for example, through statistical methods or sensitivity analyses.

**Table 2:** Good Epidemiology Practice (GEP) Criteria for Pesticide Exposure Assessment Using Biomonitoring, Personal Monitoring, and Environmental Sampling Data.

Category	Criteria for Causal Inference and Quantitative Risk Assessment	Comments
Study Protocol	An a priori protocol/study plan with defined mechanistic hypotheses is required, including reference to a thorough Standard Operating Procedure (SOP) that details sampling protocols and internal quality control. Health outcomes being investigated in the study plan should consider biologically plausible mechanisms. The protocol should include all details regarding study design and implementation. Note any deviations. While all details of the protocol will not be included in a journal article, the documents should be available on request and submitted for any studies being considered for pesticide registration and policy-making.	▪ Formal quality programs may be followed, such as the International Organization for Standardization (ISO). See WHO [17] Good Laboratory Practice (GLP) guidance for further information.
Validity and Reliability of Sampling	Exposure assessed via the same methods and within the same timeframe across all groups. Samples collected under controlled conditions; contamination assessed (e.g., via blanks analysis). Contamination from storage materials, the matrix (e.g., external contamination on toenail samples), and other sources fully considered and controlled via equipment selection, cleaning, etc. All glassware, reagents, organic solvents, and water should be checked for possible interfering contaminants. For some environmental samples, the available methods may not be well standardized, such as wipe sampling for dust exposure. In these cases, the researcher must conduct research to fill any knowledge gaps (e.g., best wipe material), and conduct method validation to optimize the methods [22].	▪ With regard to time frame, while not all samples will necessarily be taken at the same time (e.g., studies with rolling enrollment), the researcher must evaluate whether pesticide concentrations may have changed over time (e.g., due to environmental degradation, changing pesticide use) and assess how any changes may impact results. ▪ For biological samples that are time-varying (within a person) due to chemical metabolism or other factors, particularly within a single day or across weeks, the researchers must assess and either standardize sample collection across participants or adjust/stratify results to account for differences. ▪ In instances where timing affects results, stratification or other methods for evaluating inconsistencies across measures may be utilized [3]. ▪ For wipe sampling, consult relevant guidance (e.g., Battelle 2007 [22]).
Exposure Window	The exposure window of the chosen biomarker/metric reflects the time period during which the exposure could have effects relevant to the outcome of interest. It considers induction and latency and is based on biological and clinical pathways.	▪ Not applicable to case-control studies. For a developmental endpoint, the metric should capture the exposure that occurred during the period of fetal development associated with that effect (e.g., for congenital heart defects, exposure occurs during cardiac development). Similarly, for cancer, the exposure must precede the diagnosis by a sufficient latency period (typically, at least 5 years for hematologic neoplasms and 10 years for epithelial neoplasms).
Time Integration	The biomarker or personal/environmental sampling data correspond(s) to the most relevant exposure metric (e.g., average, cumulative, peak), based on the studied outcome and known biological variability.	
Specificity	Metric is specific to the exposure of interest. For biological samples, the biomarker must be the parent compound of interest or a toxicologically relevant metabolite that represents an internal dose that is well correlated with external exposure. The toxicokinetics of the parent compound in the body are understood and have been considered in choosing the metric and designing the sampling protocol.	▪ For example, dialkylphosphates, the urinary metabolites of organophosphates (OPs), are not specific to individual OPs, so they are not informative regarding specific OPs [23]. ▪ For personal monitoring and environmental exposures, several exposure metrics may be needed to provide sufficient information. Confirmatory tests can be conducted to ensure positive identification of the exposure of interest [18].
Sensitivity	Metric is sensitive (i.e., measurable down to a low limit of detection that is low enough to detect chemicals in a sufficient percentage of the samples to inform the causal or research question). There must also be high confidence in the ability of the instruments used to provide the needed level of sensitivity.	
Validity and Reliability of Analytical Methods	The analytical test methods have been validated (e.g., by assessment of repeatability within a laboratory and reproducibility of the method at multiple laboratory sites). In the case of a novel test, sufficient information must be provided regarding the within-laboratory validation, and any uncertainties should be discussed. Reliability should be assessed via intraclass correlation coefficient (ICC) or similar assessment.	
Number of Samples and Replicates	A sufficient number of replicates per sample is required to ensure data validation and assessment of data variability. For personal monitoring and environmental samples, ensure there are a sufficient number of total samples for the specific detection limit and/or to achieve sufficient power for the necessary statistical analysis (see example under comments).	▪ As noted in US EPA guidance for regulatory risk assessment, if, for example, a frequency of detection limit of five percent is used, “then at least 20 samples of a medium would be needed (i.e., one detect in 20 samples equals a five percent frequency of detection)” [24]. ▪ If using ProUCL for environmental assessment, a minimum of 4–5 samples are required.
Consideration of Matrix Effects	Adjust exposure estimate, as needed, based on the matrix and any matrix effects during sample processing. Both adjusted and unadjusted concentrations and results should be reported. Matrix effects must be investigated in the early phase of studies and used to inform the methods chosen and/or to put sample treatment remedies in place. Investigations can include comparing the response of an analyte in a standard solution and in a post-extraction spiked sample (matrix-matched standard). Preventative measures such as correcting for analyte losses during pre-treatment, may also be performed [25].	▪ For example, adjust urine measurements for creatinine (for dilution) or blood measurements for lipid content (for associations between chemicals stored in fat and where lipids may be confounders or covariates).
Sample Storage and Stability	Verify the stability of the substance of concern in the samples, given the matrix, storage conditions, and duration of storage. Samples may have some known losses, but differences between low and high exposures can be assessed.	▪ For many biological samples, storage at a range of −80 to −130 degrees Celsius is preferred [26]; deviation of this general standard must be justified. Samples that have undergone thawing and refreezing must be evaluated against spiked samples subjected to the same conditions [2]. Fluctuations in storage conditions should be avoided.
Reporting Requirements	The study protocol should be made publicly available. Details regarding the consideration and fulfillment of the above criteria (e.g., all sample collection, handling, processing, and storage; evidence of sample stability; analytical methods; method sensitivity, specificity, validity, and reliability; and Quality Assurance/Quality Control [QA/QC] procedures) should be reported. Limits of detection and limits of quantification should be stated for the target analyte, and the proportion of samples at or below these values should be recorded. Any deviations from the SOP/protocol and justifications for such deviations should be reported. While some of these details may need to be omitted for peer-reviewed publication, the information should be provided in supplemental material or made available upon request.	▪ To determine concentrations most representative of potential exposures, results above and below the limit of quantification should be considered together. Use one-half the limit of quantification as a proxy concentration if there is reason to believe the chemical is present below the limit of quantification, or use the limit of quantification value itself if there is reason to believe the concentration is closer to this value. Only use a value of zero if there is specific information indicating that the chemical is not likely to be present in a sample [24].

GEP = Good Epidemiology Practice; WHO = World Health Organization.

**Table 3:** GEP Criteria for Pesticide Exposure Assessment Using Questionnaires, Interviews, and Expert Record Review.

Category	Criteria for Causal Inference and Quantitative Risk Assessment	Comments
Study Protocol	An a priori study protocol must include standard and detailed specifications for developing questionnaires and conducting interviews; training personnel; extracting, coding, and processing data; keeping records and storing data; and quality assurance (QA) and quality control (QC) procedures that minimize the potential for bias and human error.	
Exposure Window	1. The exposure metric should be assessed in the time period during which the exposure could have effects relevant to the outcome of interest. The selection of the exposure window should consider induction and latency and should be based on biological and clinical pathways.	▪ Not applicable to case-control studies. For a developmental endpoint (e.g., congenital heart defects), the exposure metric should capture the exposure that occurred during the period of fetal development associated with that effect (e.g., fetal cardiac development). For cancer, the exposure must precede the diagnosis by a sufficient period of time (typically, at least 10 years).
Study Population	The study population should be representative of the target population with regard to the exposure distribution (i.e., it should capture the entire exposure range).	
Design of Self-administered and Interview Questionnaires	The questions should be clear and simple so as to avoid ambiguity and enhance recall. Ideally, questionnaires should be designed with multiple ways of obtaining a specific piece of information, to allow for an internal cross-check of the validity of the provided answers.	▪ For example, when assessing occupational exposures, workers should be asked about factors that are easily recalled, such as tasks, raw materials, equipment, and processes [27]. Avoid leading questions, such as, “Did you ever feel ill after using the pesticide product?”
	3. The questions should be sufficiently comprehensive and detailed to address specific research questions. For quantitative risk assessment, the questions should capture ordinal or semi-quantitative exposure information.	▪ For example, the questions should capture multiple exposure metrics (e.g., exposure frequency, exposure time, exposure intensity, time since first exposure, time since last exposure) rather than a dichotomous exposure status (i.e., yes/no, ever/never).
Blinding	Blinding of study participants and researchers should be implemented, if possible, to reduce the potential for information bias. Particularly for retrospective studies, exposure must be assessed independent of outcome.	▪ The study participants should be blinded to the specific research question (i.e., the exposure of interest). ▪ For retrospective studies, the person(s) conducting the interviews or reviewing records/questionnaires should be blinded to the outcome status of the study participants, if possible.
Validation of Exposure Assessment Methods	Self-reported exposures from questionnaires and interviews should be validated by objective records or measurements. For quantitative risk assessment, self-reported exposure information needs to validated against biomonitoring or environmental sampling data, ideally in a subset of the study population. For record review, the expert needs to be sufficiently qualified and rely on environmental measurements to determine the relative rankings of the study participants with regard to the exposure. For quantitative risk assessment of occupational exposures, a valid job-exposure matrix, based on environmental sampling data, should be constructed. The environmental sampling should capture the temporal changes and variations in the exposures by task.	▪ For example, self-reported use of pesticides can be validated with purchasing/inventory records, biomonitoring data, or personal/environmental exposure monitoring. ▪ Self-reported use of pesticides can be combined with Geographic Information Systems (GIS) methods to develop surrogate exposure estimates, but due to inability to assess fine spatial variations, GIS can introduce uncertainty or error into exposure estimates, and approaches should be validated on multiple datasets [28,29,30,31]. ▪ For assessing occupational exposures by record review and/or job-exposure matrix, the expert should have relevant experience and credentials (i.e., certified industrial hygienist).
Reporting Requirements	The study protocol should be made publicly available. Details regarding personnel training and credentials, questionnaire development, computer software employed, data extraction and processing, and methods used to estimate exposure should be reported. Any deviations from the study protocol and justifications for such deviations should be reported.	

GEP = Good Epidemiology Practice; Underlining indicates criteria that are needed for quantitative risk assessment.

**Table 4:** GEP Criteria for Assessing Dietary Exposures to Pesticides Using Environmental Sampling and Questionnaires/Interviews/Record Review.

Category	Criteria for Causal Inference and Quantitative Risk Assessment	Comments
Residue in Food (Environmental Sampling)		
Study Protocol and QA/QC Procedures	An a priori protocol/study plan is required, including reference to a thorough Standard Operating Procedure (SOP) that details sampling protocols and addresses internal quality control. The protocol should include all the details regarding the design and implementation of the study. Any deviations should be noted. While all details of the protocol will not be included in a journal article, the documents should be available on request and submitted for any studies being considered for pesticide registration and policy-making.	▪ Formal quality programs may be followed, such as the International Organization for Standardization (ISO). See WHO [17] guidance for further information. ▪ In total diet studies (TDSs), the necessary processing and preparation of foods creates a risk of contamination or chemical losses; as such, QA methods are particularly important for these types of studies [32].
Representative Food Sampling	The selection of foods for sampling must include all types of foods that are generally consumed to assess total dietary exposure. Several batches or lots of each food must be sampled to determine the ranges of residue levels. Alternatively, bulk samples from multiple batches/lots can be used to assess average pesticide residue levels. If relying on existing samples (e.g., a market basket food survey, FDA pesticide residue monitoring program), the researchers must verify that the food sampling meets the criteria outlined here. When using a market basket (MB, also known as a TDS approach), the researcher must follow general guidance outlined by WHO et al. [33], which includes specifications for sampling and processing methodology.	▪ See WHO et al. [33] for further information.
Validity and Reliability of Sampling	Exposure assessed via the same methods and within the same timeframe across all groups. Samples collected under controlled conditions; contamination assessed (e.g., via blanks analysis). Contamination from storage materials, the matrix (e.g., external contamination on toenail samples), and other sources fully considered and controlled via equipment selection, cleaning, etc. All glassware, reagents, organic solvents, and water should be checked for possible interfering contaminants. For MB or TDS methods, preparation and analysis of food as consumed is critical because preparation techniques, including peeling, washing, and heating, affect pesticide levels.	▪ With regard to timeframe, while not all samples will necessarily be taken at the same time (e.g., studies with rolling enrollment), the researcher must evaluate whether pesticide concentrations may have changed over time (e.g., due to environmental degradation, changing pesticide use) and assess how any changes may impact results. For biological samples that are time-varying (within a person) due to chemical metabolism or other factors, particularly within a single day or across weeks, the researchers must assess and either standardize sample collection across participants or adjust/stratify results to account for differences. ▪ In instances where timing affects results, stratification or other methods for evaluating inconsistencies across measures may be utilized [3]. ▪ For MB and TDS methods, consult guidance by WHO et al. [33] and FDA [34,35].
Specificity	Metric is specific to the exposure of interest. The residue measured must be the parent compound of interest or a toxicologically relevant metabolite/degradation product that represents an internal dose that is well correlated with external exposure.	
Sensitivity	Metric is sensitive (i.e., measurable down to a limit of detection that is low enough to estimate the chemical in a sufficient percentage of the samples to inform the causal or research question). There must also be high confidence in the ability of the instruments used to provide the needed level of sensitivity.	▪ For TDS/MB surveys, pooled samples are usually used. Chemical concentrations may be altered in a pooled sample (concentrated or diluted). Therefore, the analytical methods used must be of a higher sensitivity compared with those used for food compliance monitoring.
Validity and Reliability of Analytical Methods	The analytical test methods must be suitable for the food type (e.g., raw food versus processed food, consideration of lipid/water content of the food) and the substance of interest. The analytical test methods have been validated (e.g., by assessment of repeatability within a laboratory and reproducibility of the method at multiple laboratory sites). In the case of a novel test, sufficient information must be provided regarding the within-laboratory validation, and any uncertainties should be discussed. Validity should be assessed via intraclass correlation coefficient (ICC) or similar assessment.	
Number of Samples and Replicates	Ensure there are a sufficient number of total samples for the specific detection limit and/or to achieve sufficient power for the necessary statistical analysis. For screening TDS surveys, 20–30 samples may be sufficient. For refined analyses, as many as 200–300 samples may be needed [33]. A sufficient number of replicates per sample is required to ensure data validation and assessment of data variability.	▪ See WHO et al. [33] for further information.
Consideration of Matrix Effects	Adjust exposure estimate, as needed, based on the matrix. Both adjusted and un-adjusted concentrations and results should be reported.	▪ For example, measurement in food using Liquid Chromatography-Mass Spectrometry (LC-MS) is complicated by compound and matrix-dependent response suppression or enhancement, otherwise known as matrix effect (see Niessen et al. [25]).
Sample Storage and Stability	Verify the stability of the pesticide residue in the samples, given the matrix, storage conditions, and duration of storage. Samples may have some known losses, but differences between low and high exposures can be assessed.	▪ Stability of the substance of concern (e.g., the pesticide residue) in fresh or stored food must be verified. For example, recovery analysis using spiked samples should be employed or established prior to analysis. Storage conditions and duration should be reported.
Reporting Requirements	SOPs and the study protocol should be made publicly available. Details regarding the consideration and fulfillment of the above criteria (e.g., all sample collection, handling, processing, and storage; evidence of sample stability; analytical methods; method sensitivity, specificity, validity, and reliability; and QA/QC procedures) should be reported. Any deviations from the SOP/protocol and justifications for such deviations should be reported. While some of these details may need to be omitted for peer-reviewed publication, the information should be provided in supplemental material or made available upon request.	
Food Consumption (Questionnaires, Interviews, Record Review)		
Study Protocol	An a priori study protocol must include standard and detailed specifications for developing questionnaires (or using validated questionnaires) and conducting interviews; training personnel; extracting, coding, and processing data; keeping records and storing data; and quality assurance (QA) and quality control (QC) procedures that minimize the potential for bias and human error.	
Exposure Window	1. The exposure metric should be assessed in the time period during which the exposure could have effects relevant to the outcome of interest. The selection of the exposure window should consider induction and latency and should be based on biological and clinical pathways.	▪ Not applicable to case-control studies. For a developmental endpoint (e.g., congenital heart defects), the exposure metric should capture the exposure that occurred during the period of fetal development associated with that effect (e.g., fetal cardiac development). For cancer, the exposure must precede the diagnosis by a sufficient period of time (typically, at least 10 years).
Time Integration	The assessment of dietary intake should aim for habitual or long-term consumption of foods. For 24-hour dietary recall and food diaries, a sufficient and representative number of days should be used to estimate habitual consumption.	▪ Because pesticide residues are typically present in foods at very low concentrations, long-term cumulative or cumulative average exposures are generally the most relevant metrics for any potential health effects. ▪ For 24-hour recall and food diaries, multiple days are needed to sufficiently capture temporal variations in diet (e.g., week days vs. weekends; seasons).
Study Population	The study population should be representative of the target population with regard to the consumption distribution (i.e., include the range of consumption).	
Design of Self-administered and Interview Questionnaires	The questions should clear and simple so as to avoid ambiguity and enhance recall. The questions should be sufficiently comprehensive and detailed to address specific research questions. For quantitative risk assessment, the questions should capture ordinal or semi-quantitative exposure information.	▪ For 24-hour dietary recall, the interviewer should probe for information on food types, preparation methods, portion sizes, etc. ▪ Self-administered questionnaires should include foods generally consumed and culturally specific to the target population. The questionnaires should also include questions regarding consumption behaviors (e.g., frequency, preparation methods, and portion size).
Blinding	Blinding of study participants and researchers should be implemented, if possible, to reduce the potential for information bias. Particularly for retrospective studies, exposure must be assessed independently of outcome.	▪ The study participants should be blinded to the specific research question (i.e., the foods/pesticides of interest). ▪ For retrospective studies, the person(s) conducting the interviews or reviewing questionnaires/records should be blinded to the outcome status of the study participants, if possible.
Validation of Dietary Assessment Methods	Dietary records/food diaries are the gold standard for dietary assessment. Food frequency questionnaires and 24-hour dietary recalls should be validated against dietary records.	▪ Study participants should undergo training to ensure their dietary records are properly conducted and can provide robust information on food consumption.
Reporting Requirements	The study protocol should be made publicly available. Details regarding personnel training and credentials, questionnaire development, computer software employed, data extraction and processing, and methods used to estimate exposure should be reported. Any deviations from the study protocol and justifications for such deviations should be reported.	

FDA = Food and Drug Administration; GEP = Good Epidemiology Practice; QA/QC = Quality Assessment/Quality Control; WHO = World Health Organization; Underlining indicates criteria that are needed for quantitative risk assessment.
